# The Role of Communication in Romantic Attachment and Relationship Satisfaction: A Dyadic Longitudinal Study

**DOI:** 10.1111/jmft.70127

**Published:** 2026-03-13

**Authors:** Audrey‐Ann Lefebvre, Audrey Brassard, Mireille Jean, Marie‐Ève Daspe, Marie‐France Lafontaine, Katherine Péloquin

**Affiliations:** ^1^ Department of Psychology University of Sherbrooke Sherbrooke Québec Canada; ^2^ Department of Psychology University of Montréal Montréal Québec Canada; ^3^ School of Psychology University of Ottawa Ottawa Ontario Canada

**Keywords:** attachment insecurity, communication, couples, relationship satisfaction, stress

## Abstract

Researchers and clinicians note that romantic attachment insecurities, negative communication, and stressors interact in ways that gradually undermine relationship satisfaction over time. Grounding clinical models in empirical findings is crucial. This longitudinal study examined the mediating role of communication patterns in associations between romantic attachment insecurities and relationship satisfaction, while accounting for stressful life events. Path analyses conducted with 263 couples over a year revealed that attachment insecurities were indirectly associated with lower relationship satisfaction in both partners via greater use of demand/withdraw and demand/demand communication patterns. Attachment avoidance was indirectly associated with lower satisfaction in both partners via the withdraw/withdraw communication pattern. Results indicated differences according to dyad gender and revealed that stressful life events played a moderating role in the associations between attachment insecurities and communication patterns. The findings provide support for the theoretical underpinnings of both attachment‐based and communication‐based couple interventions, highlighting their clinical value.

## 1 Introduction

For many adults, romantic relationships represent the most important connection in their lives, and the quality of this relationship is a key determinant of both physical and psychological health (Slatcher and Schoebi 2017). Previous studies have shown that relationship satisfaction, a subjective evaluation of relationship quality at a given time, tends to decline over time for many couples (Joiner et al. 2024; Williamson and Lavner 2020). Both clinical and theoretical perspectives emphasize the dynamic interplay between individual vulnerabilities and relational processes in undermining relationship satisfaction. Among these, Emotionally Focused Couple Therapy (EFCT; Johnson 2020) highlights the central role of attachment insecurities and negative communication patterns in relationship distress, notably the pursuer–withdrawer dynamic reflected in the dysfunctional communication patterns. Similarly, the Vulnerability‐Stress‐Adaptation (VSA) model (Karney and Bradbury 1995; McNulty et al. 2021) underscores the importance of considering enduring qualities (attachment), dyadic adaptation processes such as communication, and stressors, as they jointly shape satisfaction. Together, these frameworks provide a coherent rationale for examining attachment insecurities and dysfunctional communication patterns simultaneously as explanatory factors of later relationship satisfaction, yet no previous longitudinal study has done so in both partners. Considering both partners is crucial, as EFCT and the VSA model emphasize the combined contribution of each partner. Providing empirical support for clinical models, such as EFCT, is essential to validate their theoretical foundations and better inform the development and application of evidence‐based interventions. This dyadic study aimed to examine the mediating role of dysfunctional communication patterns in the longitudinal links between attachment insecurities and both partners' relationship satisfaction, while considering partners' stressful life events.

## 2 Attachment Theory and Relationship Satisfaction

Attachment theory provides a conceptual framework for understanding the formation and maintenance of adult romantic relationships. This theory posits that children develop internal working models of the self and others based on how their caregiver (attachment figure) responds to their needs (Bowlby 1982). When caregivers respond consistently and sensitively to a child's needs, the child is more likely to develop a secure attachment, whereas a lack of availability or responsiveness increases the risk of developing attachment insecurities. These internal representations tend to remain relatively stable into adulthood, where the romantic partner becomes the primary attachment figure after approximately 2 years. Romantic attachment is commonly conceptualized along two continuous dimensions: anxiety and avoidance. Attachment‐related anxiety is characterized by a negative self‐view of being unlovable, with the attachment system activating in response to real or perceived relationship threats, often leading to hypervigilance to signs of rejection and an excessive need for reassurance and closeness with a partner. Attachment‐related avoidance is characterized by a negative view of others as untrustworthy, with the attachment system deactivating when the individual feels discomfort with closeness or perceives a threat to their autonomy, leading them to suppress or minimize negative emotions and create distance with their partner (Mikulincer and Shaver 2016).

Insecurely attached individuals tend to hold negative expectations of others, viewing them as potentially rejecting (anxiety) or intrusive (avoidance). This may lead to pessimistic views of their romantic relationship (Li et al. 2023) and is ultimately associated with both partners' lower relationship satisfaction (Kimmes et al. 2015; Mikulincer and Shaver 2016). Although cross‐sectional links between attachment and relationship satisfaction have been well documented (Candel and Turliuc 2019), only a few longitudinal studies have shown that higher levels of attachment insecurities are associated with a decline in relationship satisfaction over time (e.g., Fitzpatrick and Lafontaine 2017; Saavedra et al. 2010). However, the explanatory mechanisms behind these links remain understudied. Couple communication may be a key pathway through which attachment insecurities undermine relationship satisfaction, as insecure individuals tend to engage in dysfunctional communication patterns (Arseneault et al. 2024; Dugal et al. 2021) that are associated with reduced relationship satisfaction (Schrodt et al. 2014).

## 3 Couple Dysfunctional Communication Patterns

Experiencing conflict is a common and expected aspect of romantic relationships. However, it is the strategies that both partners use to resolve conflicts, not only the occurrence of conflict, that play a critical role in diminishing their relationship satisfaction (Joel et al. 2020). Communication patterns are adaptive processes enacted by both partners during interactions to manage stressors or disagreements (Karney and Bradbury 1995). Individual conflict strategies can be distinguished as a demanding stance, which involves requesting change, criticizing, or blaming, and a withdrawing stance, which involves remaining silent, evading confrontation, or distancing oneself. These strategies combine to create systemic dyadic patterns, three of which are detrimental: the demand/withdraw pattern (one partner demands while the other withdraws), the demand/demand pattern (both partners demand change), and the withdraw/withdraw pattern (both partners avoid confrontation; Christensen and Sullaway 1984).

The few longitudinal studies conducted on the demand/withdraw pattern have shown its relative stability – or chronicity – over time (Kurdek 2005; Noller et al. 1994), suggesting that some couples are particularly prone to use maladaptive behaviors during conflict. Past research has shown that individuals high in attachment insecurities are more likely to engage in the three dysfunctional communication patterns examined in this study (Arseneault et al. 2024; Dugal et al. 2021). Individuals with higher anxiety are more likely to engage in demanding behaviors to seek reassurance in relationship‐threatening conflicts (Allison et al. 2008), but they may also withdraw from conflicts to avoid rejection or accommodate their partner's needs (Bonache et al. 2019; Mikulincer and Shaver 2016). In contrast, individuals with higher avoidance are more likely to withdraw when their partner makes demands or seeks closeness. Nonetheless, their tendency to bottle up anger, resentment, and frustration (Brassard et al. 2014; Mikulincer and Shaver 2016) sometimes results in expressions of blame and criticism. Since both attachment insecurities can be associated with demanding and withdrawing stances, gaining a clearer understanding of how different dysfunctional communication patterns undermine relationship satisfaction is essential to guide clinical interventions aimed at preventing dissatisfaction in insecurely attached partners.

Several cross‐sectional studies have demonstrated that the demand/withdraw communication pattern is associated with lower levels of relationship satisfaction (e.g., Jarnecke et al. 2016; Knobloch‐Fedders et al. 2014; Schrodt et al. 2014). In contrast, the demand/demand and withdraw/withdraw patterns have received limited empirical attention. Yet, although individual behaviors that characterize demanding (e.g., criticism, blame) and withdrawing (e.g., passivity) positions have been shown to undermine both partners' satisfaction (Jarnecke et al. 2016; Knobloch‐Fedders et al. 2014), it is crucial to examine the dyadic patterns that emerge when these strategies combine, as they may have particularly detrimental effects. Most studies on communication patterns have focused on individuals or heterosexual couples (e.g., Arseneault et al. 2024; Jarnecke et al. 2016). A study suggested that women more often demand, and men withdraw (Christensen and Shenk 1991), whereas another showed that demand/withdraw occurs regardless of couple gender (Holley et al. 2010). Given these contradictory findings, examining whether results vary by dyad gender composition is essential. Findings from McNulty et al. (2021) also suggest that to fully understand how dysfunctional communication undermines satisfaction, it is essential to adopt a longitudinal and dyadic perspective that considers both partners' behaviors. Their study also highlights the importance of accounting for each partner's stress levels to capture how attachment shapes conflict behaviors that may erode satisfaction.

## 4 Role of Stressful Life Events

Dysfunctional communication patterns go beyond individual strategies, reflecting systemic interactional dynamics that inform clinical and theoretical models. Stress is an essential factor to consider, as it shapes how partners engage in these dynamics. Over time, couples are likely to encounter various stressors (e.g., accidents, financial issues) that require them to adapt together. However, navigating these challenges can be difficult, as stress has been found to increase the likelihood of conflict occurrence (Neff and Karney 2017) and is associated with poorer problem‐solving abilities (Buck and Neff 2012; Neff and Karney 2009). Williamson et al. (2013) found that individuals experiencing higher stress levels reported greater use of negative communication strategies (e.g., contempt, hostility) and lower relationship satisfaction. Similarly, a longitudinal study by Nguyen et al. (2020) showed that increases in couples' use of negative communication strategies were associated with decreased relationship satisfaction among men, but only when men were exposed to a greater number of stressors. The VSA model (Karney and Bradbury 1995), supported by a recent meta‐analysis of 10 longitudinal dyadic studies (McNulty et al. 2021), suggests that stressors (e.g., work demands, financial constraints) modulate how individual vulnerabilities – personal characteristics that each partner brings into the relationship – are associated with their adaptive processes (e.g., communication patterns). It is also recognized that in response to stressful events, highly anxious individuals seek partner support, whereas highly avoidant individuals downplay distress and rarely do so (Simpson and Rholes 2017). Despite growing evidence that stress can undermine relationship satisfaction, no study has simultaneously examined how stress modulates the longitudinal links between attachment insecurities and relationship satisfaction through dysfunctional communication patterns.

## 5 Objectives and Hypotheses

This dyadic and longitudinal study aimed to examine the dyadic and indirect links between romantic attachment insecurities (anxiety, avoidance) and relationship satisfaction through dysfunctional communication patterns, while accounting for the moderating role of stress, in adult couples. Figure S1 in the supplementary material summarizes the VSA and dyadic models underlying the hypotheses. Our first hypothesis (H1) postulates that an individual's attachment insecurities would be related to greater use of dysfunctional communication patterns as reported by both partners (demand/withdraw, demand/demand, withdraw/withdraw). Our second hypothesis (H2) suggests that an individual's attachment insecurities would be indirectly related to both their own and their partner's lower relationship satisfaction a year later, through both partners' greater use of dysfunctional communication patterns (actor and partner effects). Based on the VSA model, our third hypothesis (H3) postulates that stress would accentuate the positive association between attachment insecurities and dysfunctional communication patterns (actor and partner effects). To address potential differences and overcome prior research limitations, this study exploratorily examines whether indirect associations varied by dyad gender.

## 6 Methods

### 6.1 Participants and Procedure

This project was part of a larger study on couples' behaviors and emotions. A sample of 263 Canadian couples was recruited from the general population through advertisements on social media. Participants interested in enrolling in the study were directed to an eligibility questionnaire to provide their contact information. To be eligible, couples had to be at least 18 years old, have been in a relationship for at least 2 years, live together, and be able to read and understand French. A member of the research team contacted interested couples by phone to confirm their eligibility and explain the study procedures. Couples then received an email with a personalized link to access the consent form and baseline questionnaires (T1). Each partner completed the online questionnaires individually via the secure Qualtrics platform, which took 45 to 60 min and was administered five times (every 3 months) over a year. To support participant retention, regular reminders were sent via email at each assessment point. From T2 to T5, the questionnaires included a relationship status question to confirm that participants were still in a relationship with their partner. Couples who experienced separation during the study were no longer eligible to participate. Of the 263 couples that completed the baseline questionnaire (T1), at least one partner completed the questionnaire at T2 for 254 couples (96.58%), at T3 for 234 couples (88.97%), at T4 for 219 couples (83.27%), and at T5 for 217 couples (82.51%). Each partner received financial compensation of CAN$10 for each completed assessment. This study was approved by the university's ethics committees.

More than half of the participants (53.0%) identified as female, 43.5% as male, 3.2% as another gender identity (e.g., gender fluid, non‐binary, two‐spirits), and 0.2% did not respond. Participants' ages ranged from 20 to 76 years (*M* = 32.51, SD = 9.27). Participants (73.8%) identified as heterosexual, 8.0% as bisexual, 6.5% as homosexual, 4.4% as pansexual, 2.9% as questioning, 2.1% as queer, 0.6% as asexual, and 1.9% did not respond. Most participants were of Canadian origin (86.3%), and 35.1% of participants had at least one child (ranging 1–4). Concerning education, most participants (65.4%) completed a university degree, and half of them (52.7%) reported an annual income of less than CAN$50,000. Most participants (68.3%) were employed either full‐time or part‐time, 22.6% were students (full‐time or part‐time), 6.5% were unemployed or on sick/parental leave, and 2.7% were retired or homemakers. Couples had been together for an average of 7.8 years (ranging 2–33). Most couples were mixed‐gender (*n* = 220), and 16% (*n* = 42) were gender‐similar or diverse: 26 were female–female, 2 were male–male, and 14 included at least one partner identifying with another gender.

### 6.2 Measures

Participants completed a socio‐demographic questionnaire to gather personal information (e.g., age, gender, occupation). Validated questionnaires used in this study were administered to participants in their French versions at each assessment point.

#### 6.2.1 Romantic Attachment

Both attachment insecurities (anxiety and avoidance) were measured using the 12‐item short version of the Experiences in Close Relationships Scale (ECR‐12; Lafontaine et al. 2016). Participants indicated their level of agreement on a seven‐point Likert scale ranging from 1 (strongly disagree) to 7 (strongly agree). Total scores were calculated by averaging the items for each subscale, with higher scores indicating higher levels of attachment anxiety or avoidance. The ECR‐12 has been validated with five Canadian samples, including French‐Canadian couples from the general population, and demonstrated good internal consistency for attachment‐related anxiety (*α* = 0.78 to 0.87) and avoidance (*α* = 0.74 to 0.83; Lafontaine et al. 2016). Cronbach's values at T1 in the present study were 0.86 (anxiety) and 0.84 (avoidance) for P1, and 0.87 (anxiety) and 0.80 (avoidance) for P2.

#### 6.2.2 Dysfunctional Communication Patterns

The demand/withdraw, demand/demand, and withdraw/withdraw communication patterns were measured using the original 35‐item version of the Communication Patterns Questionnaire (Christensen and Sullaway 1984). These items assessed the three communication patterns by asking participants to rate both their own behaviors and those of their partner during problem situations on a nine‐point scale ranging from 1 (very unlikely) to 9 (very likely). Dyadic scores for each pattern were calculated by averaging these ratings across both partners' assessments, with higher scores indicating more frequent use of the pattern by couples. Cronbach's alpha coefficients obtained from a sample of couples ranged from 0.81 to 0.86 (Crenshaw et al. 2017). This study's Cronbach's alpha at T2 ranges from 0.72 to 0.85.

#### 6.2.3 Relationship Satisfaction

Relationship satisfaction was measured using the four‐item abbreviated version of the Dyadic Adjustment Scale (DAS‐4; Sabourin et al. 2005). Participants responded to the items on a six‐ or seven‐point scale. The score is calculated by summing the items, with a higher score indicating greater relationship satisfaction. The predictive validity for relationship dissolution of the DAS‐4 is supported by a longitudinal study in which participants were assessed twice over a 30‐month period. The DAS‐4 shows good internal consistency (*α* = 0.84, Sabourin et al. 2005). Cronbach's alpha at T5 was 0.73 for P1 and 0.78 for P2 in this study.

#### 6.2.4 Stress

The number of experienced stressors was measured using 32 items from the Survey of Life Events (Bradbury 1990). Participants indicated life events that occurred during the 2 years preceding the start of the study (T1), as well as those that occurred between each assessment point. The scale includes events related to work, finances, health, the couple and personal life, and relationships with close ones. The score used in the present study was the number of events that occurred between T1 and T2, with higher scores indicating greater exposure to stressors. This interval was chosen based on the VSA model, as stress during this period may activate attachment and shape couple communication. No psychometric properties are available, as it is a list of unrelated events.

### 6.3 Data Analysis Strategy

Descriptive and pairwise correlation analyses were performed using SPSS 30 software to examine participants' characteristics and links between the study variables. According to Little's MCAR test, the pattern of missing data was not completely random, *χ* ^2^(26) = 88.197, *p* < 0.001. Participants who did not complete the T5 relationship satisfaction measure differed from completers on baseline characteristics, such as higher attachment avoidance, lower relationship satisfaction, more stressful life events, and shorter relationship duration.

Dyadic mediation analyses using path analysis grounded in the Actor‐Partner Interdependence Mediation Model (Ledermann et al. 2011) were conducted in Mplus. These analyses account for the non‐independence of couple data and allow for the simultaneous evaluation of actor effects (e.g., the link between one's attachment and one's satisfaction) and partner effects (e.g., the link between one's attachment and partner's satisfaction), as well as indirect effects through intermediary variables (i.e., dyadic communication patterns). Given the mixed sample of same‐ and mixed‐gender couples, dyads were composed of partner 1 (P1) and partner 2 (P2), without distinguishing by the participants' gender. Because dyadic scores were used for communication patterns, the demand/withdraw pattern could manifest twice within a couple. As the dyads were indistinguishable, only the P2demands/P1withdraws configuration is reported. Missing data were processed using the Full Information Maximum Likelihood (Enders and Bandalos 2001) estimator in Mplus 8.11. Maximum likelihood robust estimation was used to account for univariate non‐normality in certain variables (Cole and Maxwell 2003). To ensure a more accurate estimation of the associations of interest, a series of covariates (e.g., relationship length, number of children, couple therapy) were included in the statistical models, given their significant links with satisfaction in previous research (Hadden et al. 2014; Kowal et al. 2021). The model showing the best overall fit to the data, with or without covariates, was selected.

A non‐parametric bootstrapping procedure (Preacher and Hayes 2008) with 10,000 samples was used to assess the significance of direct and indirect links between attachment insecurities (T1) and relationship satisfaction (T5), through communication patterns (T2), by calculating 95% confidence intervals around the estimates. Communication patterns at T2 were used to maximize statistical power and examine their link with later satisfaction. To assess the unique mediating contribution of each dyadic communication pattern in the links between attachment insecurities and satisfaction, three separate models were estimated for each pattern due to their high intercorrelations. Given that the demand/demand and withdraw/withdraw patterns are symmetrical, coefficients were constrained to be equal for P1 and P2, yielding actor‐only effects. In contrast, the demand/withdraw pattern is asymmetrical, and coefficients were estimated freely for each partner. Multiple‐group invariance analyses were conducted for each model to examine differences based on couples' gender composition. Two groups were created: mixed‐gender couples, consisting of one partner identifying as a man and the other as a woman, and gender‐similar/diverse couples, consisting of either two men, two women, or couples including at least one partner identifying with a different gender. To assess the moderating effects of stress on the links between attachment insecurities at T1 and communication patterns at T2, the communication patterns scores were simultaneously regressed on the predictor, the moderator, and their interaction term. Interactions were decomposed at three levels of the moderator – low (−1 SD), average (0 SD), and high (+1 SD) – only when the interaction term reached marginal significance (i.e., *p* < 0.10; Hayes 2022). The adjustment of each proposed model to the data was assessed with four indices: a non‐significant chi‐square value, a Comparative Fit Index (CFI) value of 0.95 or more, a Root Mean Square Error of Approximation (RMSEA) value of less than 0.06, and a Standardized Root Mean Square Residual (SRMR) value of less than 0.08 (Kline 2023).

## 7 Results

### 7.1 Preliminary Analyses

Significant pairwise correlations among most variables supported testing our dyadic mediation models (see Table 1). The distribution of some variables deviated from normality, as indicated by skewness and kurtosis values exceeding 1.

**Table 1:** Descriptive analysis and pairwise correlations for the main variables.

	1	2	3	4	5	6	7
1. Attachment anxiety T1	0.06	0.12**	0.22**	0.22**	0.15**	−0.10*	0.01
2. Attachment avoidance T1	0.20**	0.16**	0.29**	0.23**	0.28**	0.03	0.09
3. Demand/withdraw T2	0.22**	0.29**	—	0.68**	0.59**	−0.12**	0.16**
4. Demand/demand T2	0.22**	0.23**	0.68**	—	0.56**	−0.03	0.16**
5. Withdraw/withdraw T2	0.15**	0.28**	0.59**	0.56**	—	−0.07	0.15**
6. Relationship satisfaction T5	−0.06	−0.10*	−0.12**	−0.03	−0.07	0.45**	−0.04
7. Stressful events T2	0.13**	0.08	0.16**	0.16**	0.15**	−0.04	0.31**
*M*	3.44	2.03	2.56	2.32	2.70	16.54	2.74
SD	1.52	1.03	1.22	1.34	1.16	3.19	2.36
Skewness	0.355	1.411	0.948	1.472	0.675	−1.148	0.991
Kurtosis	−0.819	2.359	0.277	2.367	−0.010	1.280	0.898

*Note:* Correlations between variables within the same partner are shown below the diagonal. Correlations across partners are displayed above the diagonal. The diagonal entries indicate cross‐partner correlations for identical variables. Dysfunctional communication patterns are assessed as dyadic variables, shared across both partners.

Abbreviations: P1, partner 1; P2, partner 2.

* *p* < 0.05;

** *p* < 0.01.

### 7.2 Main Analyses

#### 7.2.1 Demand/Withdraw Communication Pattern

The first model testing the mediating role of the P2demands/P1withdraws pattern suggests that it played a significant role in the associations between attachment and relationship satisfaction measured 1 year later. The model without covariates showed a better fit (and was more parsimonious): *χ* ^2^(6) = 5.129, *p* = 0.527, CFI = 1.000, SRMR = 0.026, RMSEA = 0.000, 90% CI [0.000, 0.073]. To ensure that the results were not attributable to the initial assignment of partners to the P1 and P2 positions, a second model was tested in which partners were randomly reassigned to these roles. This second model showed a good fit and supported the replication of the results: *χ* ^2^(6) = 5.131, *p* = 0.527, CFI = 1.000, SRMR = 0.026, RMSEA = 0.000, 90% CI [0.000, 0.073]. We also tested the model, examining the P1demands/P2withdraws communication pattern, which yielded similar results. Therefore, only the results from the P2demands/P1withdraws pattern are presented.

**Figure 1:**
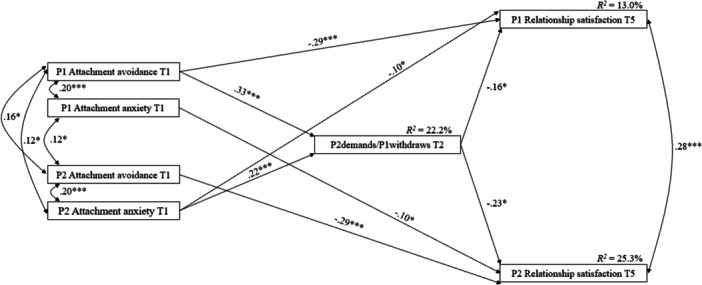
Associations among attachment insecurities, P2demands/P1withdraws, and relationship satisfaction (*N* = 263 couples). All paths between variables were tested. Only significant standardized regression coefficients are presented. P1, partner 1; P2, partner 2; T1, baseline; T2, 3 months later; T5, 12 months later. *p < 0.05, ***p < 0.001.

Figure 1 displays standardized path coefficients and explained variance. Significant direct prospective negative associations were found between participants' attachment avoidance at T1 and their own relationship satisfaction at T5 and between participants' attachment anxiety at T1 and their partner's relationship satisfaction at T5. Table 2 presents the significant indirect longitudinal associations between attachment insecurities and relationship satisfaction through dysfunctional communication patterns. Results revealed that an individual's attachment avoidance at T1 was positively associated with a pattern in which they withdraw while their partner demands at T2, which in turn was associated with their own and their partner's lower relationship satisfaction 1 year later. Results also showed that P2 attachment anxiety at T1 was associated with higher use of P2demands/P1withdraws communication pattern at T2, which in turn was associated with their own and their partner's lower relationship satisfaction 1 year later.

**Table 2:** Significant indirect associations between attachment insecurities and relationship satisfaction via communication patterns.

Model	Path	*B*	SE	*p*	95% CI
Model 1	*Actor effect*				
	P2 anxiety → P2D/P1W → P2 satisfaction	−0.051	0.022	0.020	−0.095, −0.006
	P1 avoidance → P2D/P1W → P1 satisfaction	−0.054	0.025	0.031	−0.105, −0.004
	*Partner effect*				
	P2 anxiety → P2D/P1W → P1 satisfaction	−0.035	0.017	0.036	−0,072, 0.000
	P1 avoidance → P2D/P1W → P2 satisfaction	−0.078	0.028	0.006	−0.113, −0.019
Model 2	*Actor and partner effects (constrained)*				
	Anxiety → D/D → Own satisfaction	−0.040	0.018	0.028	−0.075, −0.004
	Avoidance → D/D → Own satisfaction	−0.037	0.014	0.006	−0.064, −0.010
Model 3	*Actor and partner effects (constrained)*				
	Avoidance → W/W → Own satisfaction	−0.041	0.014	0.005	−0.070, −0.012

Abbreviations: D/D, demand/demand; P1, partner 1; P2, partner 2; P2D/P1W, P2demands/P1withdraws; W/W, withdraw/withdraw.

Multiple‐group invariance analyses revealed a significant difference between mixed‐gender couples and gender‐similar/diverse couples, *χ*²(15) = 32.67, *p* = 0.005. Specifically, in the gender‐similar/diverse couples' group only, participants' attachment anxiety at T1 was directly and negatively associated with both their own and their partner's relationship satisfaction 1 year later. Indirect links were significant only in the mixed‐gender couples' group pattern, where both attachment insecurities at T1 were related to both partners' relationship satisfaction 1 year later through P2demands/P1withdraws communication pattern measured at T2. No indirect associations were significant for gender‐similar/diverse couples' group.

To test the moderating role of stressful events, eight interaction terms (own and partner attachment at T1 × number of stressful events between T1 and T2) were added to predict communication patterns at T2. Two significant interaction effects were found. Significant moderations are illustrated in the online supplemental materials. First, P2 number of stressful events moderated the link between P2 attachment avoidance and P2demands/P1withdraws pattern, *b* = 0.14, SE = 0.06, *p* = 0.010. Simple slope analyses showed that P2 attachment avoidance was positively associated with P2demands/P1withdraws pattern only when P2 exhibited a high number of stressful events, *b* = 0.21, SE = 0.07, *p* = 0.002. However, P2 attachment avoidance was not associated with P2demands/P1withdraws pattern when P2 exhibited low (*b* = −0.08, SE = 0.09, *p* = 0.383) or average (*b* = 0.07, SE = 0.05, *p* = 0.214) number of stressful events (see Figure S2). Second, P1 number of stressful events moderated the link between P2 attachment anxiety and P2demands/P1withdraws pattern, *b* = −0.12, SE = 0.07, *p* = 0.074. P2 attachment anxiety was positively related to P2demands/P1withdraws pattern when P1 exhibited low (*b* = 0.33, SE = 0.08, *p* < 0.001) and average (*b* = 0.21, SE = 0.06, *p* < 0.001) number of stressful events, but not when it was high (*b* = 0.09, SE = 0.10, *p* = 0.342; see Figure S3).

#### 7.2.2 Demand/Demand Communication Pattern

**Figure 2:**
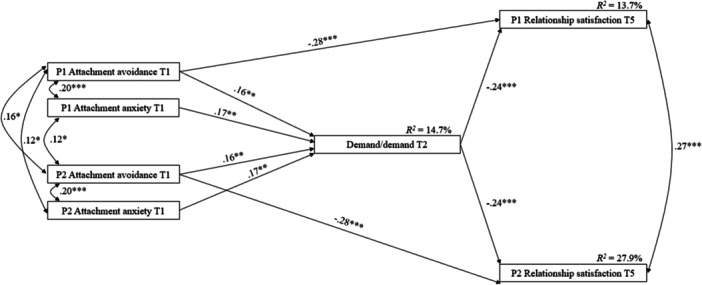
Associations among attachment insecurities, demand/demand, and relationship satisfaction (*N* = 263 couples). All paths between variables were tested. Only significant standardized regression coefficients are presented. P1, partner 1; P2, partner 2; T1, baseline; T2, 3 months later; T5, 12 months later. *p < 0.05, **p < 0.01, ***p < 0.001.

The second model tested the mediating role of the demand/demand communication pattern at T2 in the links between attachment at T1 and relationship satisfaction measured 1 year later. The model without covariates showed better fit: *χ* ^2^(9) = 5.541, *p* = 0.785, CFI = 1.000, SRMR = 0.029, RMSEA = 0.000, 90% CI [0.000, 0.046]. Results revealed a significant negative direct link between one's attachment avoidance at T1 and their own satisfaction 1 year later (see Figure 2). Also, both attachment insecurities (anxiety and avoidance) at T1 were related to greater use of the demand/demand communication pattern at T2, which, in turn, was related to both partners' lower satisfaction 1 year later (see Table 2).

Multiple‐group invariance analyses revealed a significant difference between mixed‐gender couples and gender‐similar/diverse couples, *χ*²(12) = 25.575, *p* = 0.012. Only in the gender‐similar/diverse couples' group was participants' attachment anxiety at T1 directly and negatively associated with their partner's satisfaction 1 year later. Indirect associations between attachment insecurities (anxiety and avoidance) at T1 and both partners' satisfaction 1 year later, via the demand/demand pattern at T2, emerged only in the mixed‐gender couples' group. In the gender‐similar/diverse couples' group, no significant indirect associations were found.

The model, including the eight interaction terms between attachment insecurities at T1 and the number of stressful life events occurring between T1 and T2, revealed one significant moderation effect, *b* = 0.19, SE = 0.06, *p* = 0.001. Simple slopes analyses revealed that P1 attachment avoidance was positively related to demand/demand pattern when P1 exhibited a high number of stressful events, *b* = 0.26, SE = 0.08, *p* = 0.001. However, P1 attachment avoidance was no longer related to demand/demand pattern when P1 exhibited low (*b* = −0.12, SE = 0.09, *p* = 0.211) and average (*b* = 0.07, SE = 0.06, *p* = 0.257) number of stressful events (see Figure S4).

#### 7.2.3 Withdraw/Withdraw Communication Pattern

**Figure 3:**
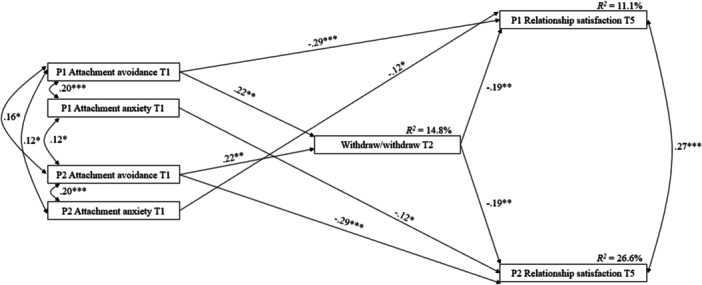
Associations among attachment insecurities, withdraw/withdraw, and relationship satisfaction (*N* = 263 couples). All paths between variables were tested. Only significant standardized regression coefficients are presented. P1, partner 1; P2, partner 2; T1, baseline; T2, 3 months later; T5, 12 months later. *p < 0.05, **p < 0.01, ***p < 0.001.

The third model examining the withdraw/withdraw communication pattern also showed a better fit without covariates: *χ* ^2^(9) = 9.671, *p* = 0.378, CFI = 0.995, SRMR = 0.035, RMSEA = 0.017, 90% CI [0.000, 0.073]. Findings indicated two significant direct links between attachment at T1 and relationship satisfaction 1 year later (see Figure 3). Participants' attachment avoidance at T1 was associated with their own relationship satisfaction, whereas participants' attachment anxiety at T1 was related to their partner's relationship satisfaction, with both outcomes assessed 1 year later. Indirect effect results (see Table 2) revealed that both partners' attachment avoidance at T1 was related to greater use of the withdraw/withdraw communication pattern at T2, which, in turn, was related to lower relationship satisfaction for both partners 1 year later.

Multiple‐group invariance analyses revealed one significant difference between mixed‐gender couples and gender‐similar/diverse couples, *χ*²(12) = 28.213, *p* = 0.005. As in the previous model, partners' attachment anxiety at T1 was directly and negatively related to their partners' relationship satisfaction 1 year later, only in the gender‐similar/diverse couples.

One significant interaction effect emerged in the model testing the moderating role of stressful events between T1 and T2 in the links between attachment at T1 and satisfaction 1 year later. P2 number of stressful events moderated the link between P2 attachment avoidance and withdraw/withdraw pattern (*b* = 0.12, SE = 0.07, *p* = 0.090). P2 attachment avoidance was positively related to withdraw/withdraw pattern when P2 exhibited average (*b* = 0.25, SE = 0.07, *p* < 0.001) and high (*b* = 0.37, SE = 0.09, *p* < 0.001) number of stressful events. However, P2 attachment avoidance was no longer associated with withdraw/withdraw pattern when P2 exhibited low (*b* = 0.12, SE = 0.11, *p* = 0.261) number of stressful events (see Figure S5).

## 8 Discussion

Expanding previous studies that have primarily focused on the demand/withdraw communication pattern or relied on correlational designs (e.g., Jarnecke et al. 2016; Knobloch‐Fedders et al. 2014), this study adopted a dyadic longitudinal approach to investigate how three types of dysfunctional communication patterns could act as mechanisms linking romantic attachment insecurities to lower relationship satisfaction over time. Our findings lend empirical support for the theoretical foundations of clinical models, notably EFCT, which inform attachment‐ and communication‐based couple interventions. In addition, guided by the VSA model, this study emphasized the importance of considering the context in which relationship difficulties unfolded, encompassing both the stressful life events experienced by the partners. Couple‐specific characteristics were also considered, such as the gender composition of the dyad.

### 8.1 Romantic Attachment Anxiety

Results partially supported our hypotheses (H1 and H2), indicating that partners with higher attachment anxiety were more likely to adopt a demanding stance within the demand/withdraw and demand/demand patterns, regardless of partner strategy. This was associated with lower relationship satisfaction for both partners 1 year later. Our findings align with EFCT, which posits that demanding–pursuing interaction patterns undermine the satisfaction of both partners (Johnson 2020). This finding is also consistent with prior research showing that attachment anxiety is associated with the demanding stance (e.g., Arseneault et al. 2024; Dugal et al. 2021). Individuals with attachment anxiety, hypervigilant to relational threats, often engage in attachment system activation strategies, such as reassurance‐seeking and pursuing behaviors to increase closeness (Allison et al. 2008). Their heightened sensitivity may trigger strong emotional reactions (e.g., anger, despair) rooted in fears of rejection or abandonment, which could undermine constructive communication. As a result, their partner may be more likely to withdraw rather than seek closeness, or to adopt a demanding stance when obliged to engage in problem discussions with the anxiously attached partner (Pietromonaco et al. 2004). Over time, an anxiously attached partner may intensify their efforts to elicit a response from their partner, viewing them as the sole person capable of fulfilling their needs. The escalation of their demands – from adequate to hostile – could contribute to decreased satisfaction. Prior studies supported this dynamic, demonstrating that both expressing and receiving demanding behaviors are related to lower satisfaction (Jarnecke et al. 2016; Knobloch‐Fedders et al. 2014).

Our results extend previous research by using a longitudinal design with a large sample of 263 couples, including several gender‐similar and gender‐diverse couples. Our results also showed that indirect links between attachment insecurities and relationship satisfaction, via demand/withdraw and demand/demand patterns, were non‐significant for gender‐similar/diverse couples, which is a novel finding of this study. Although some studies reported similar use of demand/withdraw patterns across all couple types (Holley et al. 2010), others highlighted differences in conflict management, with gay and lesbian partners often showing more constructive communication than heterosexual couples (Kurdek 2005; Gottman et al. 2003). Our sample of 42 gender‐similar/diverse couples may have been too small to detect indirect effects between attachment insecurities and relationship satisfaction via communication patterns.

Beyond these indirect effects, a negative direct link remained between participants' attachment anxiety and their partner's relationship satisfaction 1 year later (except in the demand/demand model). Anxiety‐driven behaviors that anxiously attached individuals tend to adopt may undermine their partner's satisfaction, which may explain partners' lower satisfaction even when communication patterns are considered. For instance, their tendency to act jealously and their difficulty offering sensitive support may erode their partner's sense of security in the relationship. For anxiously attached individuals, sexual difficulties, such as engaging in intimacy primarily to regulate insecurity rather than to build a genuine connection, may undermine their partner's satisfaction over time (Mikulincer and Shaver 2016). Although the main results revealed a direct link between participants' attachment anxiety and their partner's satisfaction, dyadic gender differences nuanced this finding, showing it was present only in gender‐similar/diverse couples. An additional actor link between attachment anxiety and satisfaction was found in the demand/withdraw model. These links may reflect mechanisms beyond communication, consistent with the minority stress model (Meyer 2003), whereby discrimination, internalized stigma, and social rejection could undermine emotional closeness and relationship satisfaction in sexual minority couples (Frost and Meyer 2023; Song et al. 2024). In mixed‐gender couples, attachment anxiety may lower partners' satisfaction primarily through communication patterns.

### 8.2 Romantic Attachment Avoidance

Our findings supported our hypothesis (H1 and H2) and showed that when one partner exhibited higher attachment avoidance, they tended to adopt both withdrawing and demanding stances, which were in turn associated with lower relationship satisfaction in both partners. These results aligned with previous research indicating that avoidantly attached individuals often assume a withdrawing stance during conflicts (Arseneault et al. 2024; Dugal et al. 2021). These individuals tend to deactivate their attachment system during conflict to shield themselves from their partner's demands, thereby preserving their independence and emotional distance. Although this may reduce relational strain, it often leads them to withdraw from situations that require emotional closeness or vulnerability (Mikulincer and Shaver 2016), which could diminish their satisfaction over time. However, avoidantly attached individuals may adopt a demanding stance when maintaining emotional distance is no longer possible. In situations where they feel forced to discuss a problem with their partner, they may respond with criticism, blame, and defensiveness (Arseneault et al. 2024; Dugal et al. 2021). These behaviors could escalate conflict, hindering constructive communication, which could contribute to undermining satisfaction (Bretaña et al. 2022; Schrodt et al. 2014). This study extends prior research by examining the withdraw/withdraw pattern, often overlooked and rarely linked to both partners' satisfaction. EFCT also recognizes that mutual withdrawal from conflict can erode satisfaction by hindering emotional connection and attachment‐related resolution (Johnson 2020).

Beyond these indirect links, a negative direct association remained between participants' attachment avoidance and their own relationship satisfaction, which is consistent with previous studies (Fitzpatrick and Lafontaine 2017; Saavedra et al. 2010). Individuals with attachment avoidance tend to hold negative expectations regarding their partner and the relationship, often expecting unreliability and interpreting interactions through a negative lens. They may also engage in behaviors that create or maintain emotional distance, which could reinforce their negative view of their relationship (Kimmes et al. 2015; Mikulincer and Shaver 2016).

### 8.3 Stress as a Moderator

Drawing on core components of the VSA model, our study highlights the role of stress as a contextual factor shaping the links between attachment and communication. Our results partially supported our third hypothesis (H3), showing that when an individual reported a low or average number of stressful life events, their partner, when they exhibited higher attachment anxiety, tended to adopt the demanding stance in the demand/withdraw communication pattern. At these lower or moderate levels of partner stress, anxiously attached individuals may perceive their partner's withdrawal during conflict as a lack of emotional support or responsiveness. Such withdrawal could heighten anxiously attached individuals' fears and encourage greater use of demanding strategies (Simpson and Overall 2014). However, when partners reported high stress, this association became nonsignificant, suggesting that anxiously attached individuals may temper demands and adopt a more accommodating, supportive stance (Simpson and Rholes 2017).

Our findings revealed that when individuals reported an average or high number of stressors – but not when they reported low levels – their level of attachment avoidance was related to both partners' greater use of the withdrawing stance (withdraw/withdraw model). This suggests that partners might temporarily set aside conflicts to provide space and reduce relational strain when the avoidantly attached individual experiences multiple stressors, as elevated stress may deplete the resources needed to engage constructively during conflict (Neff and Karney 2017). Indeed, studies showed that partners tended to resort to withdrawal when they face stress to reduce arousal and help regain a baseline emotional and physiological state (e.g., Neff and Karney 2017; Repetti 1989). For avoidantly attached individuals, this tactic also serves to reduce exposure to intimacy and vulnerability, thereby minimizing discomfort (Mikulincer and Shaver 2016; Simpson and Overall 2014). However, our moderation analyses revealed that when individuals reported a high number of stressful life events, their level of attachment avoidance was related to greater use of the demanding stance. When withdrawal is no longer an option, individuals with attachment avoidance who are experiencing several stressors may more easily shift to a critical and blaming position (Arseneault et al. 2024; Dugal et al. 2021). Individuals with higher attachment avoidance tend to suppress negative emotions and frustrations (Brassard et al. 2014; Mikulincer and Shaver 2016), but under stress, they may have fewer resources to minimize, deny, or suppress them (Neff and Karney 2017), leading to increased defensiveness. Our results highlight stress as a key contextual factor in couple dynamics, supporting the VSA model (McNulty et al. 2021).

### 8.4 Limitations

This study was the first to test the mediating role of three dysfunctional communication patterns in the longitudinal associations between attachment insecurities and relationship satisfaction in a large, diverse couple sample. A key strength was the inclusion of core VSA components and the examination of gender‐based differences in indirect associations within the dyads. Yet, this study presents limitations that should be acknowledged. First, the sample size did not provide enough statistical power to examine, within a single model, the combined effects of all communication patterns, moderation, and gender diversity within dyads. As couples generally reported relatively high relationship satisfaction and had been together for more than 2 years, had limited cultural and sociodemographic diversity, and only a small proportion had children, this may limit the generalizability of the findings. Future studies should recruit a more culturally diverse sample of couples, including participants from clinical populations, to capture a broader range of relationship dynamics. All our measures relied on self‐reports, which are subject to social desirability and recall biases. Future research should incorporate observational or daily diary methods to better capture contextualized communication patterns and address limitations of questionnaire‐based data. The stress measure used in this study combined relationship‐specific and general stress into a single score (e.g., stressful life events), which may not have captured daily variations or context‐specific relational stress. In future research, it would be valuable to examine other mechanisms underlying the links between attachment insecurities and relationship satisfaction, such as emotion regulation or partner responsiveness (Simpson and Overall 2014).

### 8.5 Clinical Implications

The present findings provide support for the theoretical foundations of empirically validated attachment‐based couple interventions such as EFCT (Johnson 2020), as well as approaches in which communication is a primary therapeutic target, such as Cognitive‐Behavioral Therapy (CBT; Lebow and Snyder 2023). Specifically, the links between attachment insecurities and dysfunctional communication confirm key EFCT assumptions that insecure attachment underlies negative interactional cycles in couples. Moreover, communication patterns mediating the link between greater attachment insecurities and lower satisfaction highlight them as a key mechanism. This aligns with CBT models targeting maladaptive communication behaviors and emphasizes the importance of helping partners understand how their own attachment can lead to dysfunctional communication patterns that undermine satisfaction, while guiding them toward constructive strategies to meet their needs. In EFCT, therapists help couples reshape negative patterns by fostering safe expression of emotions and attachment needs instead of using blame, criticism, or withdrawal (Johnson 2020). Although communication patterns provide valuable insight, they may not fully encompass the complexity of attachment dynamics, with other processes (e.g., emotion regulation) continuing to play a key role. The moderating role of stressful life events highlights how contextual factors shape these dynamics. This underscore the need for clinicians to tailor their approach by actively considering external stressors that may affect partners' interaction patterns. Finally, differences across couple types stress the importance of systematically assessing each dyad's interaction patterns, rather than assuming that dynamics documented in mixed‐gender couples generalize to all couple configurations (Scott et al. 2019).

## Supporting information


**Figure S1:** Illustration of the VSA theoretical model and the dyadic model with the study variables. **Figure S2:** Moderating Role of P2 Number of Stressful Life Events in the Association between P2 Attachment Avoidance and P2demands/P1withdraws Communication Pattern. **Figure S3:** Moderating Role of P1 Number of Stressful Life Events in the Association between P2 Attachment Anxiety and P2demands/P1withdraws Communication Pattern. **Figure S4:** Moderating Role of P1 Number of Stressful Life Events in the Association between P1 Attachment Avoidance and Demand/demand Communication Pattern. **Figure S5:** Moderating Role of P2 Number of Stressful Life Events in the Association between P2 Attachment Avoidance and Withdraw/withdraw Communication Pattern.
