# Supplementary material for: The Role of Communication in Romantic Attachment and Relationship Satisfaction: A Dyadic Longitudinal Study
Source: J Marital Fam Ther. 2026 Mar 13;52(2):e70127. doi: 10.1111/jmft.70127 (PMC12983993; doi:10.1111/jmft.70127)

*Figure S1.* Illustration of the VSA theoretical model and the dyadic model with the study variables.

*
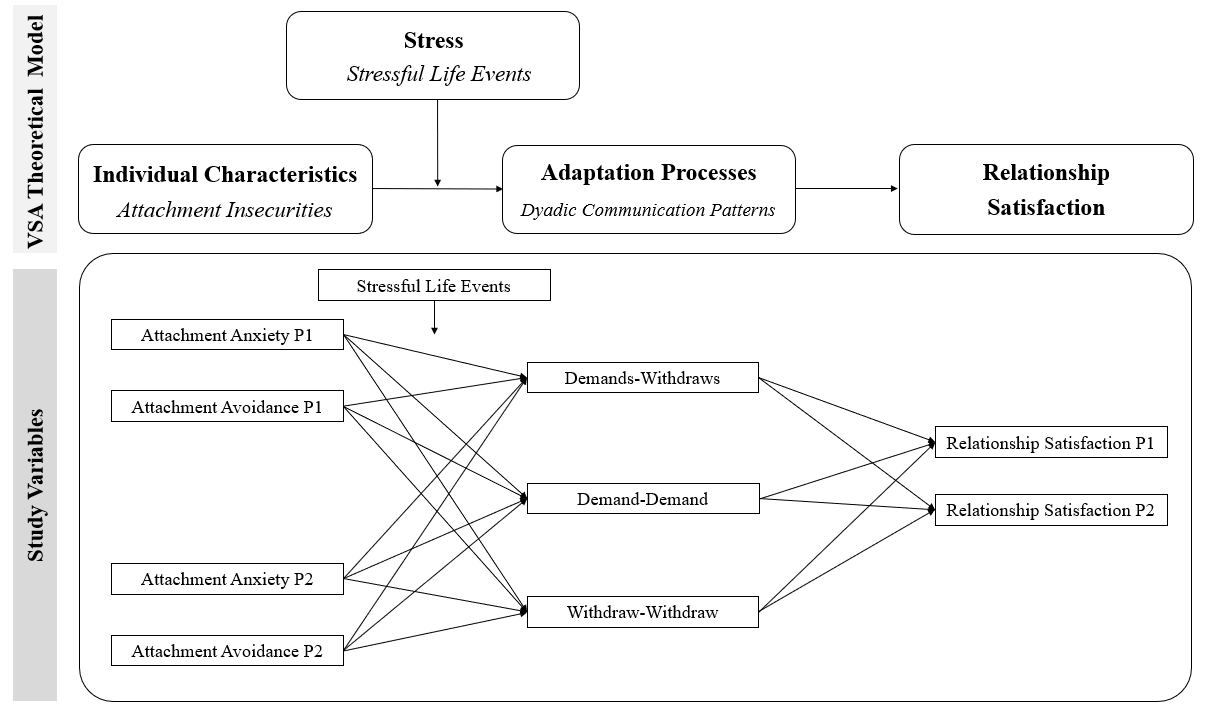
*

*Figure S2.* Moderating Role of P2 Number of Stressful Life Events in the Association between P2 Attachment Avoidance and P2demands/P1withdraws Communication Pattern.
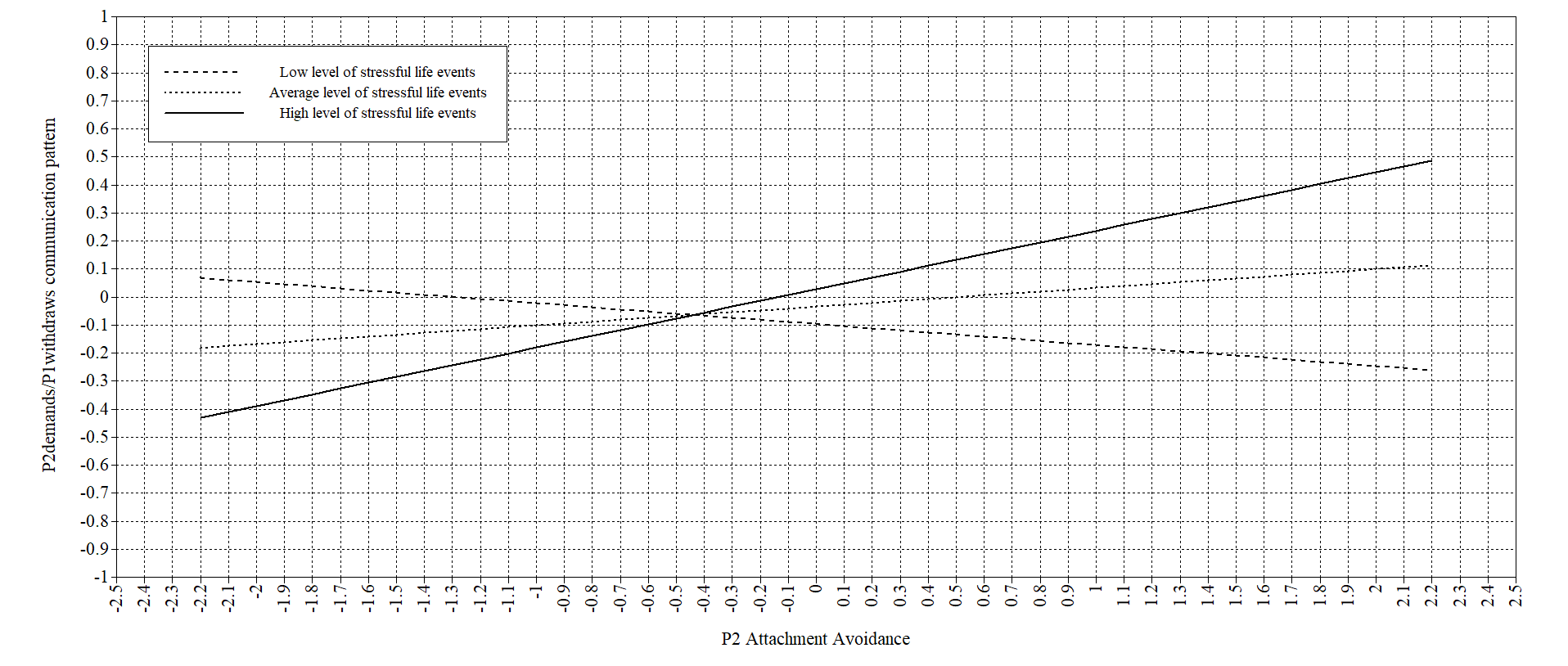


*Figure S3.* Moderating Role of P1 Number of Stressful Life Events in the Association between P2 Attachment Anxiety and P2demands/P1withdraws Communication Pattern.


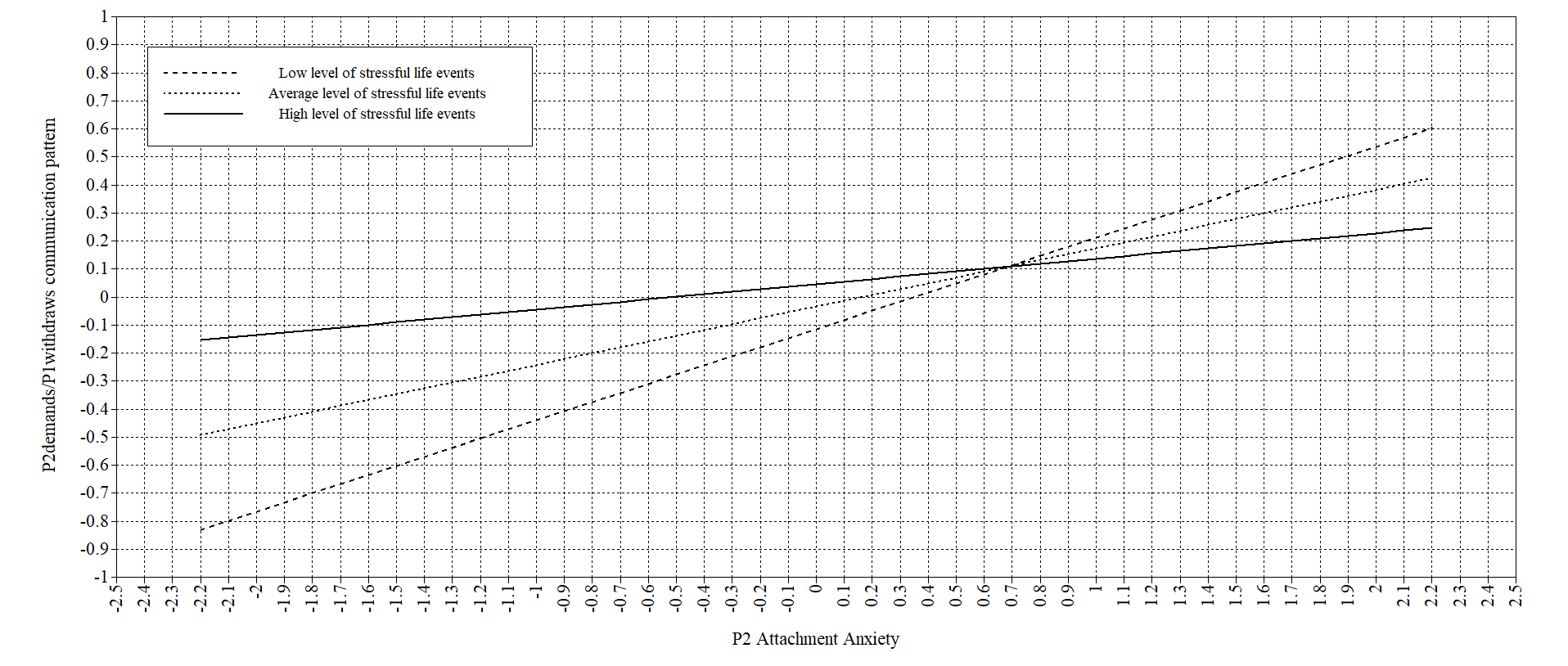


*Figure S4.* Moderating Role of P1 Number of Stressful Life Events in the Association between P1 Attachment Avoidance and Demand/demand Communication Pattern.


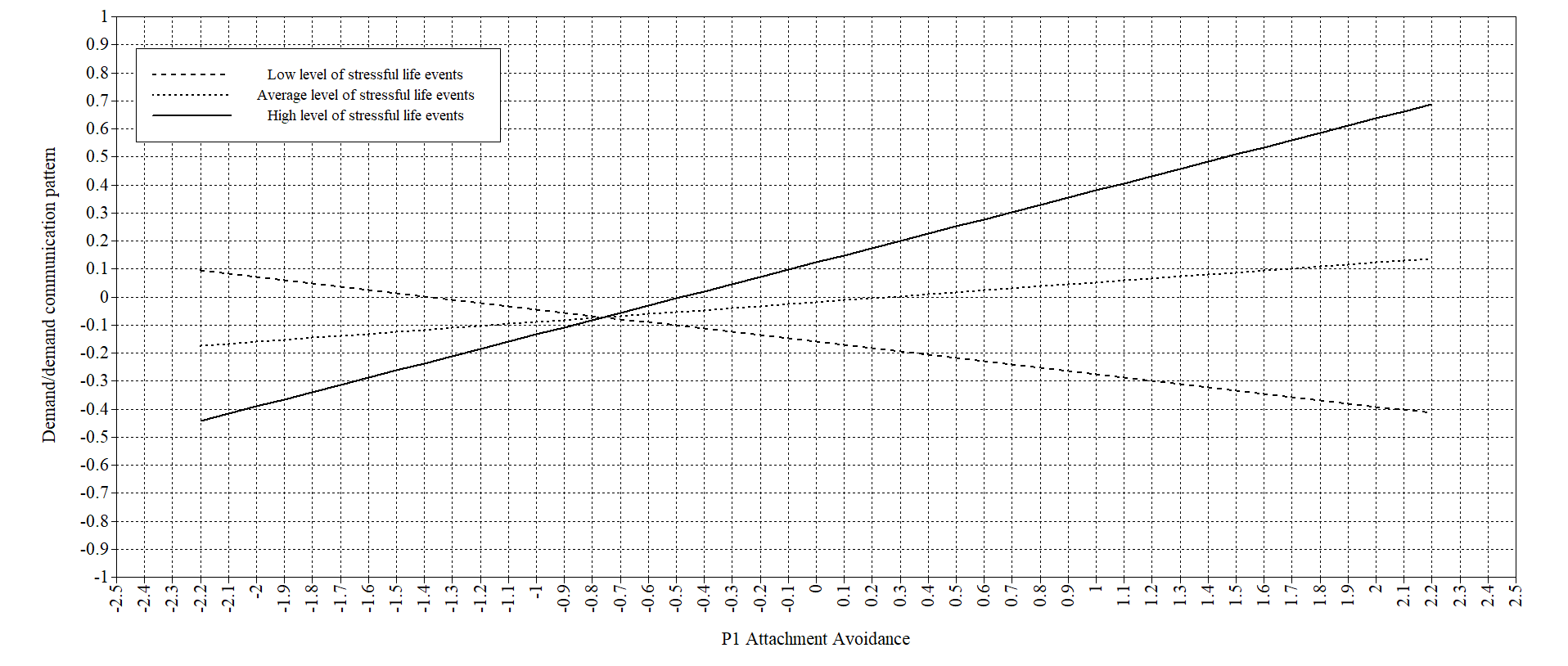


*Figure S5.* Moderating Role of P2 Number of Stressful Life Events in the Association between P2 Attachment Avoidance and Withdraw/withdraw Communication Pattern.


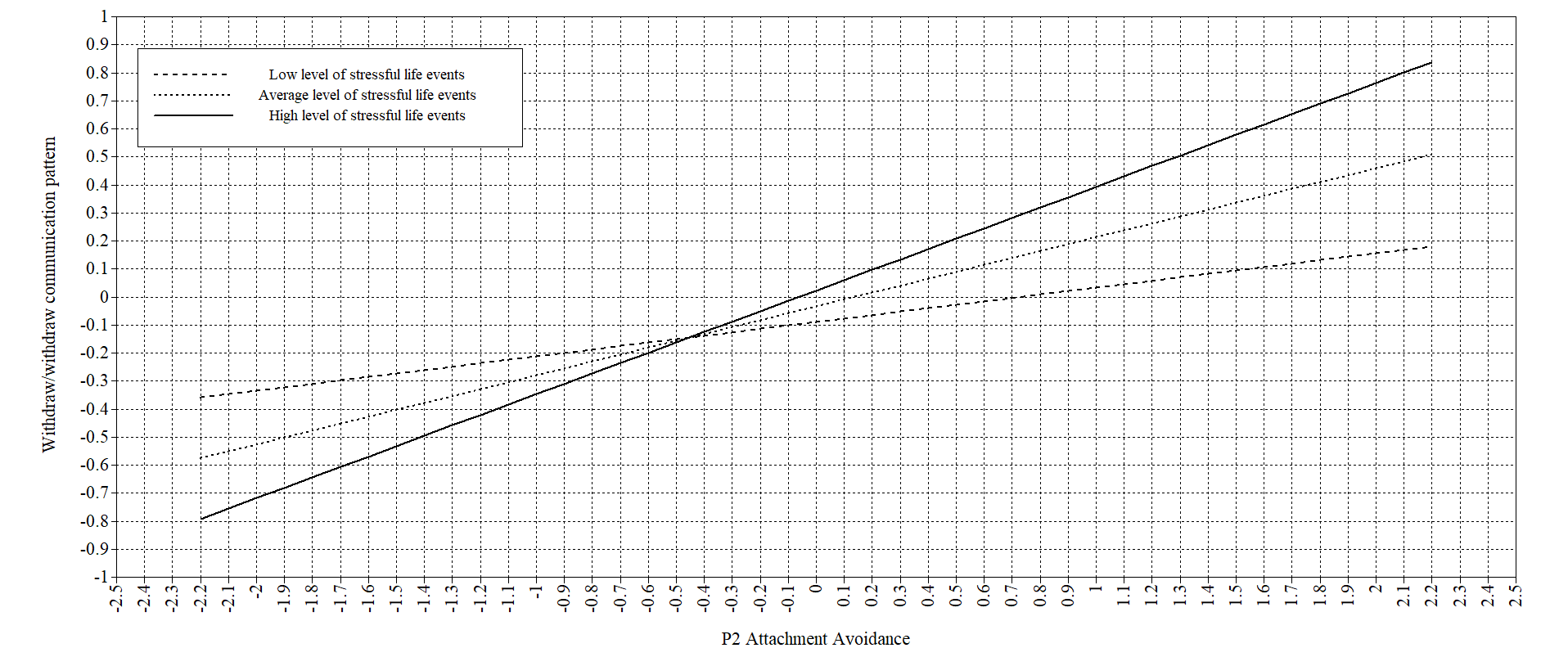

Supplement: Supplementary file 1 — Figure S1: Illustration of the VSA theoretical model and the dyadic model with the study variables. Figure S2: Moderating Role of P2 Number of Stressful Life Events in the Association between P2 Attachment Avoidance and P2demands/P1withdraws Communication Pattern. Figure S3: Moderating Role of P1 Number of Stressful Life Events in the Association between P2 Attachment Anxiety and P2demands/P1withdraws Communication Pattern. Figure S4: Moderating Role of P1 Number of Stressful Life Events in the Association between P1 Attachment Avoidance and Demand/demand Communication Pattern. Figure S5: Moderating Role of P2 Number of Stressful Life Events in the Association between P2 Attachment Avoidance and Withdraw/withdraw Communication Pattern. [file JMFT-52-0-s001.docx]
